# Use of a trephine bur and curette for minimally invasive harvesting of particulate cancellous bone and marrow from the iliac crest: a case of alveolar ridge reconstruction

**DOI:** 10.1186/s40729-015-0033-z

**Published:** 2016-01-04

**Authors:** Yukimori Isoda, Eisaku Imamura, Daisuke Ueno, Tsubasa Akaike, Yoshiki Hamada

**Affiliations:** 1 Unit of Oral and Maxillofacial Implantology, Tsurumi University Dental Hospital, Yokohama, Japan; 2 Department of Oral and Maxillofacial Surgery, Yokohama General Hospital, 2201-5 Kurogane-cho, Aoba-ku, Yokohama-shi, Kanagawa 225-0025 Japan; 3 Department of Implantology and Periodontology, Graduate School of Dentistry, Kanagawa Dental University, Yokohama, Japan; 4 Department of Oral and Maxillofacial Surgery, Tsurumi University School of Dental Medicine, Yokohama, Japan

**Keywords:** Bone graft, Particulate cancellous bone and marrow, Trephine bur, Minimally invasive surgery

## Abstract

Iliac particulate cancellous bone and marrow (PCBM) is still the most predictable autogenous graft material for vertical ridge reconstruction because of its high cell content as well as osteoinductive and osteoconductive properties. However, postoperative meralgia paresthetica, gait disturbance, pain, and bleeding have been reported following conventional harvesting from the anterior iliac crest. We present a case of minimally invasive harvesting of iliac PCBM. A short incision was made, and the iliac crest was exposed after elevation of the periosteal membrane. Only the iliac cortical bone was removed using a trephine bur to avoid perforation. PCBM was harvested with hand curettes and grafted into the vertical ridge defect. Because of the small surgical field, gait disturbance was resolved within 1 day without other postoperative complications. This technique is potentially useful for harvesting a small amount of iliac PCBM.

## Background

Recently, bone substitute materials have been used in prosthetic treatment with dental implants to reduce surgical invasion, cost, and potential resorption. These products have shown predictable results for localized defects and sinus floor augmentation [1, 2]. However, autogenous particulate cancellous bone and marrow (PCBM) is still the gold standard for extensive bone reconstructions because of the high level of osteocompetent cells for new bone formation. The ilium is the best donor site in terms of cellular quality, and the posterior ilium contains the largest amount of PCBM [3]. The disadvantage of conventional harvesting methods from the iliac crest is the increased risk of postoperative meralgia paresthetica, gait disturbance, pain, and bleeding [4–6]. To prevent these complications, less invasive procedures are required. This case report describes an alveolar ridge reconstruction with harvested PCBM from the iliac crest using a new minimally invasive technique.

## Case presentation

### Case report

This clinical report was approved by the Ethics Committee of Yokohama General Hospital, Yokohama, Japan (No. 27–002). A 42-year-old man visited the Department of Oral and Maxillofacial Surgery, Yokohama General Hospital in June 2006 with the chief complaint of pain arising from the maxillary right lateral incisor (tooth #7) and impacted canine (tooth #6). A fistula and slight swelling were observed on the alveololabial gingiva of tooth #7. No pus discharge from the fistula was found. Radiographic examination revealed apical periodontitis with root resorption of tooth #6. Furthermore, the radiolucent area extended to the labial alveolar bone of tooth #7. Because the infection had spread around the impacted tooth #6, teeth #6 and #7 were extracted under local anesthesia in August 2006. A prophylactic antibiotic^1^ was prescribed at 1 day prior to extraction.

**Fig. 1:**
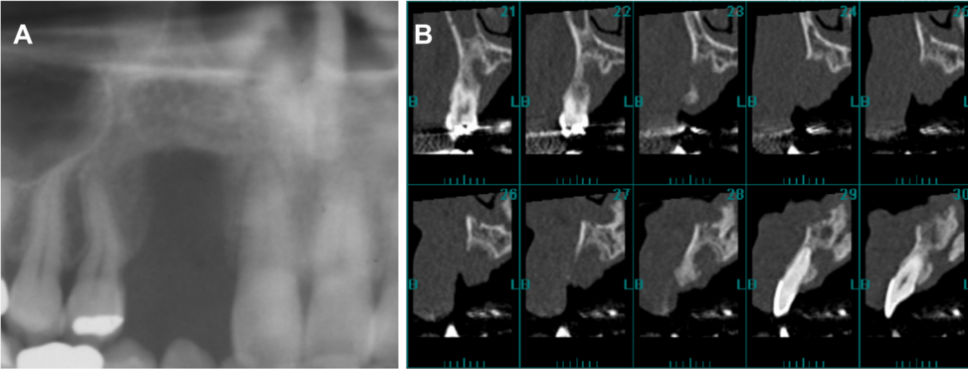
Preoperative radiographic images. **a**, **b** Panoramic radiograph (**a**) and cross-sectional CT images (**b**) show vertical bone resorption in the missing teeth region of #6 and #7

**Fig. 2:**
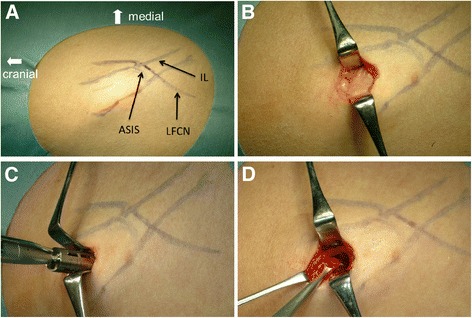
Surgical procedures for harvesting of PCBM from the iliac crest. **a** Planning of the anatomic landmarks before the incision. The inguinal ligament (*IL*), anterior superior iliac spine (*ASIS*), and lateral femoral cutaneous nerve (*LFCN*) are indicated by pyoktanin blue. **b** Preparation of the full-thickness flaps following a short incision. **c** Cutting of the cortical bone using a trephine bur. **d** Harvesting of PCBM with a surgical curette through the window in the cortical bone

**Fig. 3:**
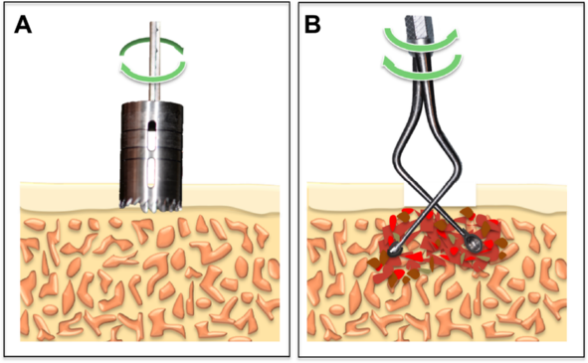
**a** Cortical bone was hollowed out using a trephine bur. **b** PCBM was gently harvested from the hollowed region using a curette

At 4 months after the extraction, the patient requested implant treatment in the missing teeth region. However, severe alveolar bone resorption was observed on radiographic images (Fig. 1). We decided to perform alveolar ridge reconstruction prior to the placement of dental implants. Under general anesthesia, we made a 15-mm skin incision and exposed the iliac crest covered with the periosteum (Fig. 2). The periosteum on the medial aspect was elevated, and a window in the cortical bone was created with a motor-driven trephine bur (8-mm diameter). About 2 g of PCBM was harvested with bone curettes (Fig. 3), before the removed cortical bone was replaced in its original position. The wound was completely closed. A pressure dressing was used for 48 h postoperatively [7].

**Fig. 4:**
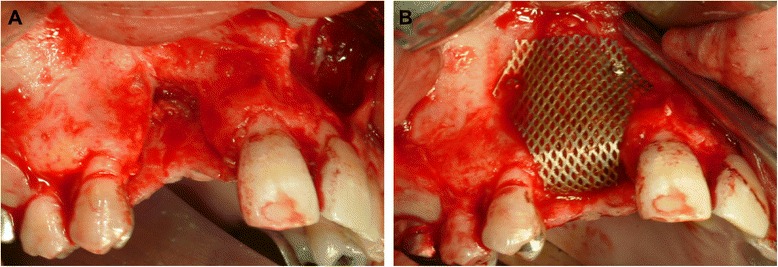
**a** Intraoral view immediately after flap elevation. A vertical bone defect without the buccal and palatal walls is seen. **b** Intraoral view immediately after PCBM grafting covered by a trimmed titanium micromesh sheet

**Fig. 5:**
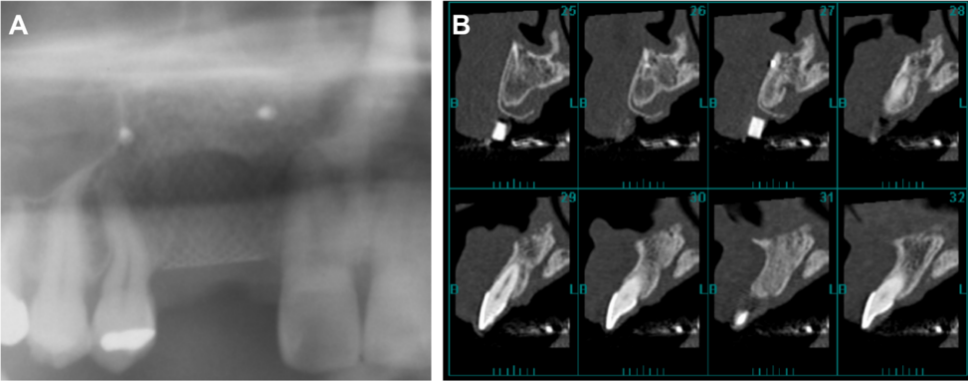
Preoperative radiographic images at 9 months after bone grafting. **a**, **b** Panoramic radiograph (**a**) and cross-sectional CT images (**b**) show significant bone regeneration in the missing teeth region of #6 and #7

**Fig. 6:**
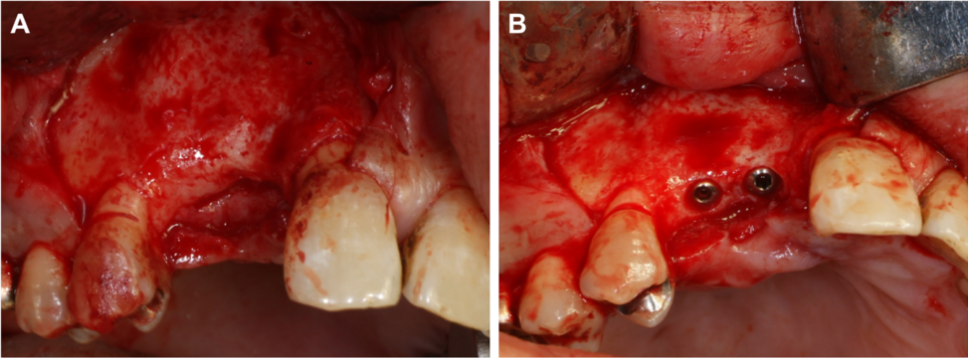
**a** Intraoral view immediately after flap elevation at 9 months after bone grafting. Significant vertical bone regeneration is observed. **b** Two dental implants were placed in the optimal sites

Following the above procedure, the recipient site was exposed after elevation of a full-thickness flap (Fig. 4a). Multiple small cortical penetrations were made, and the PCBM was closely placed on the recipient site followed by the setting of a shape-adapted titanium micromesh sheet^2^ (Fig. 4b). A periosteum-releasing incision of the flap was made, and the grafted site was completely closed by the flap using 4-0 nylon sutures. At the postoperative 2-week follow-up, a 3-mm-diameter wound dehiscence was found at the grafted site. An antibiotic^3^ was prescribed (200 mg twice-daily for 7 days). No infections were observed up to the 9-month follow-up, and CT images revealed an adequate reconstructed alveolar ridge for placement of dental implants (Fig. 5). Therefore, the titanium micromesh sheet was removed, and two dental implants^4^ of 3.5-mm diameter (lengths, 13 and 11 mm) were placed on the reconstructed alveolar ridge (Fig. 6). The implant stability quotient (ISQ) values were 77 in site #6 and 76 in site #7, as measured by an Osstell™ Mentor,^5^ indicating excellent stability at the time of implant placement [8].

**Fig. 7:**
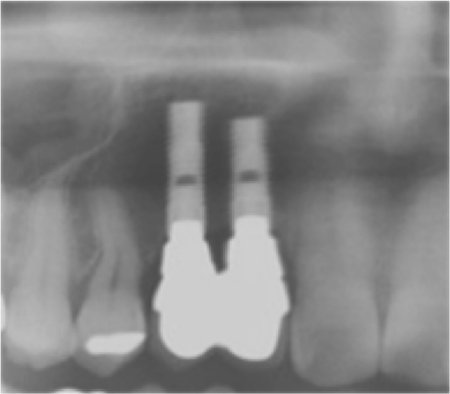
Vertical bone height is maintained at 3 years after fixation of the prosthesis

Alveolar ridge reconstruction using iliac PCBM achieved sufficient vertical bone regeneration for implant placement. At the 3-year follow-up, radiographs showed excellent results (Fig. 7).

### Discussion

The present case demonstrated a minimally invasive approach for extraoral harvesting compared with conventional methods [6]. The iliac crest is one of the most popular donor sites for major maxillofacial reconstruction, because it contains the greatest volume of PCBM and includes a rich supply of osteocompetent cells [3]. However, meralgia paresthetica and gait disturbance have been reported as major postoperative complications [4–6]. A literature review reported 0–20 % temporary and 0–5 % permanent sensory disturbances of the lateral femoral cutaneous nerve (LFCN) [9]. When the LFCN is located in the vicinity of the anterior superior iliac spine (ASIS), flap retraction causes damage, leading to dysesthesia [9]. Harvesting from the anterior iliac crest carries a risk of LFCN injury because the course of the LFCN is rarely superolateral to the ASIS. In such cases, a layer-by-layer dissection of the soft tissues can reduce the risk of nerve injury. The LFCN should be considered the most susceptible to iatrogenic injury [9]. Majkrzak et al. [10] reported that the LFCN was observed to cross the inguinal ligament at 1.4 ± 0.4 cm medial to the ASIS and traverse the inguinal ligament at 1.0 ± 0.1 cm deep to the ligament. For the best approach to the anterior ilium, a skin incision should be made parallel to and below the iliac crest by beginning at least 2 cm superior and lateral to the ASIS [9].

A recent clinical report evaluated morbidity associated with iliac crest harvesting [11]. At 1 week after harvesting, 28 and 72 % of the subjects felt severe pain and moderate pain, respectively. At 1 month after harvesting, 22 and 67 % of the subjects felt moderate pain and mild pain, respectively. The scars after harvesting were also evaluated, based on the patient satisfaction score, revealing that 22 % of the subjects felt bad about their condition [11]. Furthermore, Joshi et al. [12] reported that approximately 56 % of patients who underwent conventional iliac crest harvesting had postoperative gait disturbance for more than 2 weeks. Because gait disturbance is associated with the degree of invasiveness, a short incision can minimize the risk of complications [6]. To our knowledge, few articles have reported harvesting techniques using a trephine bur [4, 6, 13]. Burstein et al. [6] reported that the trephine bur technique can reduce incision length compared with conventional methods (2 vs. 5 cm). As a result, the use of the trephine bur technique permitted earlier ambulation and discharge from hospital [4, 6, 13]. Missiuna et al. [14] reported the morbidity associated with iliac crest harvesting using a trephine bur. They found that 69 and 100 % of the subjects were completely without pain at 1 and 8 weeks, respectively, after harvesting. Furthermore, none of the subjects reported any unpleasant signs and symptoms related to the residual scar. In another study, the use of a trephine to procure corticocancellous bone cores from the anterior iliac crest was found to carry a high risk of peritoneal perforation [15]. In our case, the trephine bur was used only for cutting of the cortical bone, and the cancellous bone was gently harvested with hand curettes. Consequently, there were no surgical complications.

Although the present technique can reduce surgical invasion, harvesting volume is limited compared with the conventional methods. When a defect is not too large, but requires graft material with high osteogenic ability, this technique is suitable for harvesting a small amount of cancellous bone. When a large amount of grafting volume is required, the conventional techniques should be selected. Currently, the most common extraoral donor sites other than the anterior iliac crest are the proximal tibia and posterior iliac crest, because both of these sites can yield a significantly greater mean volume of compressed cancellous bone than the anterior iliac crest [16]. Since hormonal as well as constitutional factors seem to be more relevant to the iliac crest than to the tibia, significantly higher bone density and volume with better osteogenic potential were observed compared with the tibia in elderly patients [17]. A disadvantage of tibial bone harvesting may be that the postoperative scar often causes cosmetic disturbance when patients wear short trousers or skirts. In contrast, the postoperative scar from harvesting of iliac bone is inconspicuous because of its location. Therefore, the properties of each harvesting methodology should be considered when extraoral harvesting is required.

The present case demonstrated significant bone regeneration in a two-wall bone defect. Because PCBM underwent remodeling and became mature bone at 9 months after grafting [18], two implants were able to be placed in the optimal site with excellent primal stability (ISQ values 77 in site #6 and 76 in site #7). It is generally accepted that implant stability can be reliably confirmed for implants with an ISQ of more than 47 [19]. The present results indicate that bone grafting using PCBM can lead to the acquisition of excellent bone quality in the grafted site even though the bone grafting was performed in a setting of advanced bone resorption.

## Conclusions

The present case report demonstrates a minimally invasive harvesting technique of PCBM from the iliac crest. The following important points need to be considered: (1) to avoid LFCN injury, a skin incision should be made parallel to and below the iliac crest by beginning at least 2 cm superior and lateral to the ASIS and (2) to minimize the risk of postoperative complications, the trephine bur should only be used for cutting of the cortical bone, and cancellous bone should be softly harvested with hand curettes after a short incision.

## Consent

Written informed consent was obtained from the patient for publication of this case report and any accompanying images.

## Footnotes

1 Cefdinir; Sawai Pharmaceutical Co. Ltd., Osaka, Japan.

2 Titanium micromesh sheet 0.1-mm thickness; M-TAM; Stryker Leibinger GmbH & Co., MI, USA.

3 Clarithromycin; Taisho Pharmaceutical Co. Ltd., Tokyo, Japan.

4 MicroThread™; AstraTech, Mölndal, Sweden.

5 Osstell™ Mentor; Integration Diagnostics Ltd., Göteborg, Sweden.
